# Heat Shock Protein 90 (Hsp90)-Inhibitor-Luminespib-Loaded-Protein-Based Nanoformulation for Cancer Therapy

**DOI:** 10.3390/polym12081798

**Published:** 2020-08-11

**Authors:** Ankit K. Rochani, Sivakumar Balasubramanian, Aswathy Ravindran Girija, Toru Maekawa, Gagan Kaushal, D. Sakthi Kumar

**Affiliations:** 1 Bio Nano Electronics Research Centre, Graduate School of Interdisciplinary New Science, Toyo University, Saitama 350-8585, Japan; ankit.rochani@jefferson.edu (A.K.R.); sivakbt@gmail.com (S.B.); aswathyrg@gmail.com (A.R.G.); maekawa@toyo.jp (T.M.); 2 Jefferson College of Pharmacy, Thomas Jefferson University, Philadelphia, PA 19107, USA; gagan.kaushal@jefferson.edu

**Keywords:** BSA nanoparticles, Hsp90, luminespib (NVP-AUY922), pancreatic cancer, breast cancer

## Abstract

Drugs targeting heat shock protein 90 (Hsp90) have been extensively explored for their anticancer potential in advanced clinical trials. Nanoformulations have been an important drug delivery platform for the anticancer molecules like Hsp90 inhibitors. It has been reported that bovine serum albumin (BSA) nanoparticles (NPs) serve as carriers for anticancer drugs, which have been extensively explored for their therapeutic efficacy against cancers. Luminespib (also known as NVP-AUY922) is a new generation Hsp90 inhibitor that was introduced recently. It is one of the most studied Hsp90 inhibitors for a variety of cancers in Phase I and II clinical trials and is similar to its predecessors such as the ansamycin class of molecules. To our knowledge, nanoformulations for luminespib remain unexplored for their anticancer potential. In the present study, we developed aqueous dispensable BSA NPs for controlled delivery of luminespib. The luminespib-loaded BSA NPs were characterized by SEM, TEM, FTIR, XPS, UV-visible spectroscopy and fluorescence spectroscopy. The results suggest that luminespib interacts by non-covalent reversible interactions with BSA to form drug-loaded BSA NPs (DNPs). Our in vitro evaluations suggest that DNP-based aqueous nanoformulations can be used in both pancreatic (MIA PaCa-2) and breast (MCF-7) cancer therapy.

## 1. Introduction

The development of drugs against breast and pancreatic cancer is a major challenge due to various back channels, such as drug efflux mechanism by p-glycoproteins (p-gp), development of drug resistance to human epidermal growth factor 2 (HER2) targeted therapy and overexpression of heat shock proteins (HSPs). Newer strategies such as mono or combination therapies that target multiple cellular pathways of cancer cells are being developed to fight against these complexities of breast and pancreatic cancer [1,2,3]. Studies suggests that development of drug-targeting HSPs is one of the most promising ways to fight a variety of cancers [4].

The microenvironment of cancer cells is extremely stressful due to acidic pH, oxidative stress, nutritional stress, phototoxic stress, hypoxic and other stress conditions. This turbulent cellular microenvironment serves as a hallmark for cancer. It has been shown that heat shock protein 90 (Hsp90) is overexpressed in cancer cells in order to buffer such stressful conditions and provide cyto-protective function to them [4,5,6]. Hsp90 is an important molecular chaperone, which has thousands of client proteins that play an important roles in the growth promotion and the survival of cancer cells under stress conditions [7]. Hence, Hsp90 works as a central hub for survival of cancer cells by interacting with a large number of unfolded or damaged essential proteins and helps in their folding by forming a multichaperone complex. As a result, inhibition of Hsp90 can hinder a large number of essential cellular pathways for cancer cells, which ultimately leads to cell death. An ansamycin analog-17-allylamino 17-demethoxygeldanamycin (17AAG)—was the first Hsp90 inhibitor that entered clinical trial against cancer by Bristol Myers Squibb. There are around fifteen molecules that have been explored as single or combination therapies. Most Hsp90 inhibitors acts by binding to the N-terminal domain that causes inhibition of the protein-folding pathway followed by activation of proteasomal degradation of client protein [8].

Being one of the most promising cancer drug targets, Hsp90 inhibitors were explored for their efficacy against breast cancer therapy. It was reported that 17AAG proved to be effective in a phase II trial for HER2 *+ve* breast cancer therapy [9]. Other Hsp90 inhibitors, like 17-Dimethylaminoethylamino-17-demethoxygeldanamycin (17DMAG), 17-Allylamino-17-Demethoxygeldanamycin Hydroquinone Hydrochloride (IPI-504), have also shown promising results towards breast cancer, pancreatic cancer and various forms of cancer treatment in single or combination therapy. This indicates that Hsp90 inhibitors can be effective therapeutic molecules against multiple cancer types. Various parent structures like resorcinol, purine analogs and others are being pursued in order to identify newer derivatives as anticancer molecules, which offers improved binding affinities towards Hsp90 proteins and are effective against various phenotypes of cancers compared to first generation ansamycin analogs [10,11].

**Figure 1:**
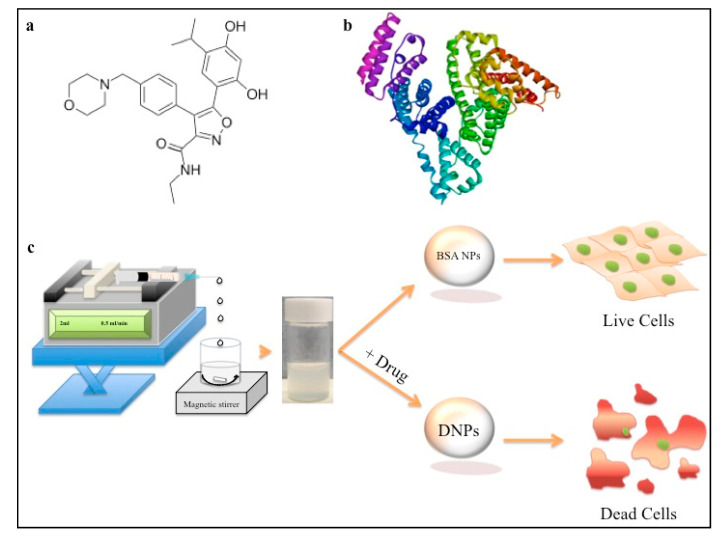
(**a**,**b**) Structure of luminespib and bovine serum albumin (BSA) (protein databank (PDB): 3V03), respectively; (**c**) schematics of synthesis and anticancer efficacy of drug-loaded BSA nanoparticles (NPs) towards cancer cell lines in comparison to native or blank BSA NPs.

Luminespib was recently introduced in the quest for finding a newer potent Hsp90 inhibitor. It is an iso-oxazole resorcinol-based synthetic Hsp90 inhibitor (as shown in Figure 1a) that was discovered with an intention to develop newer and better anticancer molecules [12]. It is was explored for its broad-spectrum anticancer activity in >10 clinical trials (clinicaltrials.gov), including estrogen receptor (ER) *+ve* and herceptine (HER) *+ve* breast cancer conditions [13,14]. The molecule was also found to be effective towards pancreatic cancer (MIA PaCa-2) cell line [15]. Hence, we believe that this single molecule may be useful for chemotherapy in multiple cancer types. Although it is a clinically explored experimental new drug molecule, luminespib remains unexplored for developing controlled delivery nanoformulations. The only formulation used for luminespib in a preclinical breast cancer model was in 60-mM lactic acid or 2.5% ethanol, 20% 50-mM tartaric acid and 77% (5% glucose in water containing 1% Tween-80) vol/vol [12,16]. Infusion of the complex formulation comprising ethanol, tartaric acid and lactic acid may lead to dose dependent toxicity. The molecule was explored in early Phase I and Phase II clinical trials against breast cancer, non-small cell lung cancer, refractory multiple myeloma, advanced solid tumors and others [17,18,19]. We believe that the development of water dispensable nanoformulations of luminespib should help to increase patient compliance.

A number of polymeric and nonpolymeric nanocarriers have been extensively explored for their potential drug formulation applications [20,21]. Polymeric nanoformulations have played an important role in developing successful nanoparticle (NP)-based anticancer therapy [7]. Polymeric nanoformulations have been used in developing novel aqueous formulation for hydrophobic Hsp90 inhibitors like 17AAG. Our lab had developed hydrophilic polymeric nanoformulations as 2-in-1 iron oxide (MNP) and 17AAG-loaded polymeric nanoformulation with the aim of combining the cytotoxicity property of 17AAG with magnetic hyperthermia in the same formulation [22]. Recently, there is a constant demand in the ideas of using various biopolymers to make better nanoformulations.

Protein-based carriers such as serum albumin have shown an intriguing potential to be utilized in nanoformulation [23,24]. Serum albumin is one of the most abundant proteins in the circulating system of a wide variety of organisms. The two most common types of albumin that are used in biomedical applications; (a) bovine serum albumin (BSA) and (b) human serum albumin (HSA). BSA shares nearly 76% sequence homology with HSA [25]. Hence, BSA is the most commonly used model protein for drug interaction studies and nanodrug delivery vehicle. Serum albumin is a major molecule in the plasma of animals, and it accounts for nearly 60% of total protein (with a concentration of 42 g dm^−3^). It consists of a single chain of 582 amino acids divided into three linearly arranged domains (I–III) [26]. The interaction of anticancer drugs with BSA has been extensively explored for developing novel formulations. Nab-paclitaxel (Abraxane) is the first albumin-stabilized nanoformulation to be approved by Food and Drug Administration (FDA) in 2005 for its efficacy in the treatment of breast cancer [27]. It was also approved by FDA for metastatic pancreatic cancer and non-small cell lung cancer. BSA–dextran–folic acid–doxorubicin nanoconjugate also showed efficacy in murine ascites hepatoma H22 tumor-bearing mice [28]. Furthermore, gemcitabine and folate gemcitabine-loaded nanoconjugates showed efficacy against pancreatic and MCF-7 breast cancer cell lines [29]. Albumin nanocarriers for 17AAG were also explored. It was found that nab-17AAG with an average size of 210 nm was shown to have considerable anticancer activity towards MX-1 breast cancer cell line [30]. The study provided positive evidence that the nab formulation of Hsp90 inhibitor could improve its efficacy, safety and stability in comparison to their surfactant and solvent-based formulations [30]. Based on the success of earlier studies, we have explored the possibility to develop BSA nanoparticles consisting of luminespib and investigated its biocompatibility and efficacy towards MCF-7 and MIA PaCa-2 cell lines.

## 2. Materials and Methods

BSA was obtained from Sigma-Aldrich (Tokyo, Japan), ethanol (99%) was procured from Wako (Tokyo, Japan); luminespib was purchased from LC laboratories (Woburn, MA, USA). Thin-layer chromatography (TLC) plates were obtained from Merck Millipore (Burlington, MA, USA). Trypan blue, trypsin EDTA (0.025%), alamar blue cytotoxicity kit and Dulbecco’s modified Eagle’s medium (DMEM) were procured from Invitrogen (Tokyo, Japan).

### 2.1. Synthesis of BSA-Luminespib Nanoconjugates

The synthesis of luminespib (drug) BSA nanoconjugates was achieved using desolvation method [31,32]. BSA protein (100 mg) was dissolved in distilled water and the pH was adjusted to around 8.0. The basic pH adjustments were performed using 0.1-M NaOH. To 10 mg of luminespib, 2 mL of absolute ethanol was added to dissolve the drug, and the (ethanolic-drug) solution was dropwise added to BSA (0.5 mL) solution at the rate of 0.5 mL/min using a syringe pump. Slow addition of drug containing ethanol solution causes formation of white suspension that indicates formation of drug–BSA conjugates. Native BSA NPs were prepared by controlled addition of ethanol to BSA solution. These drug–BSA NPs and native BSA NPs were subjected to crosslinking with 8% glutaraldehyde solution at room temperature for 4 to 5 h. The cross-linked drug-BSA nanoconjugates were subjected to washing with distilled water and centrifuged at 15,000 rpm. The collected pellet was freeze-dried and reconstituted in phosphate buffer saline (7.4 pH, PBS) for further studies.

### 2.2. Luminespib BSA Particle Characterization Studies

Specific quantity of BSA-drug nanoconjugates was filtered and analyzed for particle size, zeta potential and poly-dispersity index determination using Malvern NanoZS analyzer. The data were recorded using Zetasizer (version 2.0, Malvern Panalytical, Tokyo, Japan). All the experiments were performed in triplicates and the data were expressed as standard deviation. Surface morphology characteristics of nanoparticles were determined using scanning electron (SEM) and Transmission electron microscopy (TEM). For SEM, the sample was dropped on glass substrate, vacuum dried and coated with platinum for taking images using SEM 6600 (Hitachi, Tokyo, Japan). DNP sample was dropped on TEM hydrophilic copper grid and images were taken using JEM-2100 (Jeol, Tokyo, Japan), at 160 kV.

### 2.3. In Silico Molecular Docking

Docking calculations were carried out using Docking Server [33]. The crystal structure of BSA was downloaded from protein databank (PDB), accession number 3V03. The structure of the ligand (luminespib) with PubChem ID 135539077 was taken for carrying out the docking simulations. PM-6 partial calculations method was used for both ligand and protein. Further, the geometry for ligand was performed using MMFF94. Nonpolar hydrogen atoms were merged, and rotatable bonds were defined. Essential hydrogen atoms, Kollman united atom type charges, and solvation parameters were added with the help of AutoDock tools [34]. Affinity (grid) maps of 90 × 60 × 80 Å grid points and 0.375 Å spacing were generated using the Autogrid program (Virtua Drug, Budapest, Hungary and Scripps Research Institute, La Jolla, CA, USA). AutoDock parameter set and distance-dependent dielectric functions were used in the calculation of the van der Waals and the electrostatic terms, respectively.

Docking simulations were performed using the Lamarckian genetic algorithm (LGA) and the Solis & Wets local search method used by DockingServer GUI [35]. Initial position, orientation and torsions of the ligand molecules were set randomly. All rotatable torsions were released during docking. Each docking experiment was derived from 100 different runs that were set to terminate after a maximum of 2,500,000 energy evaluations. The population size was set to 150. During the search, a translational step of 0.2 Å, and quaternion and torsion steps of 5 were applied.

For analysis, docking results were ranked based on free energy of binding (kcal/mol), number of hydrogen bonds, polar interactions and frequency of probable binding sites. The frequency shows the percentage of the local searches with similar geometry having root mean square tolerance (rmstol) of 2 Å. The docked structures with lowest binding free energy, with maximum number of polar and hydrogen bond interactions, and with frequency of 10% or more were used for prediction of probable binding configuration. The analyzed docking file in.pdb format was downloaded and analyzed using Discovery Studio Visualizer 4.0 (Biovia, Tokyo, Japan).

### 2.4. Luminespib-BSA Interaction Studies

#### 2.4.1. Fluorescence Quenching Studies for Drug BSA Complex

Fluorescence spectroscopy measurements were carried out for a fixed concentration of BSA 12.5 µM. The drug concentration was varied from 0.0 to 12.5 µM at room temperature. Various concentrations of drug were made in 30% ethanolic PBS solution at pH 7.4. Fluorescence spectra were measured using FP6500 (Jasco, Tokyo, Japan), Jasco instrument. The excitation were kept as 280 nm. Further, at the same instrument settings we also checked the quench in fluorescence spectra for BSA–drug nanoconjugates in comparison to native BSA particle in PBS (pH 7.4) at 1 mg/mL concentration.

#### 2.4.2. UV-Vis Absorption Studies

To determine the presence of drug in the BSA NPs; we performed UV-vis. absorption studies for 1 mg/mL concentration of drug–BSA conjugates (DNPs) dispersion in PBS (pH 7.4). Here, the UV spectrum was also recorded for the plain BSA nanoparticle and free drug as control.

#### 2.4.3. Thin-Layer Chromatography (TLC)

To understand the drug interaction with BSA and formation of nab-luminespib nanoformulations, we performed TLC measurements. We spotted 10 µL of 1 mg/mL concentration of DNPs in deionized water on TLC plates and compared them to blank BSA NPs and native drug as controls. We used chloroform, methanol and concentrated NH_4_OH (80:20:1) as mobile phase. *R_f_* value was calculated for spots after checking TLC plates under UV illumination.

#### 2.4.4. X-ray Photoelectron Spectroscopy Studies (XPS)

XPS measurement was performed using Phi Quantes XPS system (Ulvac-Phi, Kanagawa, Japan). Samples were mounted on glass slide and subjected to recording for individual spectral recordings for carbon, nitrogen, oxygen and wide scan. All the measurements were made using aluminum X-ray at 55-pass energy. The XPS data were analyzed using Multipack software (Ulvac-Phi, Kanagawa, Japan).

### 2.5. Encapsulation and Drug Loading Evaluation

In our study, 1 mg of BSA-drug nanoconjugates was taken and dispersed in 1 mL of ethanol and subjected to quantitation of luminespib at 310 nm (DU^®^ 730 UV Spectrometer by Beckman Coulter, Tokyo, Japan). Drug encapsulation and drug-loading efficiency studies were performed in triplicates using the following Equations (1) and (2):

(1) $$Encapsulation\;\;efficiency\;\left(\%\right)=\frac{Amount\;of\;Luminespib\;in\;nanoparticles}{Total\;weight\;of\;Luminespib\;used\;for\;synthesis}\times 100$$

(2) $$Drug\;loading\;efficiency\;\left(\%\right)=\frac{Amount\;of\;Luminespib\;in\;nanoparticles}{Gross\;weight\;of\;nanoparticles}\times 100$$

### 2.6. Stability of DNPs

To study the stability of DNPs at ambient room temperature, nanoparticles were stored at 25 °C in glass vial. Samples were drawn to check the stability of formulation for up to seven days. Here, the particle size distribution and SEM image were used as quantitative and qualitative checkpoints, respectively.

### 2.7. In Vitro Drug Release for DNPs

In our study, 20 mg of nanoparticles were taken and dispersed in 20 mL of deionized water. The solutions were distributed in Eppendorf tubes such that each tube consisted of 1 mg/mL concentration. All the tubes were kept in a shaking incubator at 37 °C. At predetermined time intervals supernatant was taken and subjected to UV absorbance reading at 310 nm for determination of quantity of drug released. All the measurements were performed in triplicates and data were plotted as an average of the drug released at specific time points.

### 2.8. Cell Culture and In Vitro Cytotoxicity Studies

To check the cytotoxicity, we carried out culturing of MCF-7 and MIA PaCa-2 cancer cell lines. We also cultured normal mouse fibroblast cell line (L929) as control. MCF-7 and L929 cell lines were cultured in DMEM media consisting of 10% FBS and 5% penicillin/streptomycin (100 units per mL). MIA PaCa-2 was cultured in RPMI media with 10% FBS and 5% penicillin/streptomycin (100 units per mL). All the cells were incubated at 37 °C in a humidified CO_2_ environment. The confluent T-25 plates were sub cultured every third day.

MCF-7, MIA PaCa-2 and L929 cells were seeded in 96-well plates with a cell density of 5000 cells per well and cytotoxicity assay was performed with the synthesized nanoformulations using alamar blue dye. After 24 h of incubation in 96-well plates, cell lines were treated with DNPs in concentration dependent manner from 100 to 1000 µg/mL. The treated plates were subjected to measurement of fluorescence at excitation and emission wavelength as 580 and 530 nm, respectively. The measurements were made using Power scan HT, Micro plate reader, Dainippon Sumitomo Pharma, Osaka, Japan. The cell viability measurements were made for 48 h. We used Equation (3) for measurement of% cell viability. All the experiments were performed in triplicates and subjected to Student’s *t*-test for statistical significance.

(3) $$Cell\;viability\;\left(\%\right)=\frac{Asample}{Acontrol}\times 100$$

## 3. Results and Discussion

### 3.1. Synthesis, Particle Size Characterization and Encapsulation of BSA Luminespib NPs

**Figure 2:**
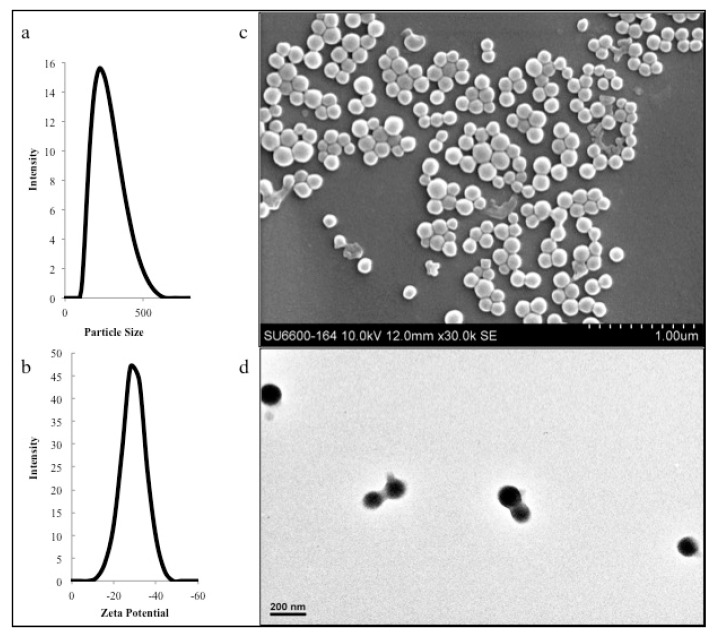
(**a**,**b**) Particle size distribution between 78 to 500 nm and zeta-potential distribution of −11.8 to −48.1 mV. The average particle size (obtained from DLS) was found to be around 222 nm and average zeta potential as around −30 mV; (**c**,**d**) SEM and TEM images of the synthesized DNPs, respectively. SEM images shows particle various sizes around ~200 nm. TEM images confirms small particle size ~100 to 150 nm. The DNP images from SEM and TEM confirmed the observation from DLS.

Synthesis of DNPs was carried out using a desolvation method wherein ethanol acts as an anti-solvent as shown in Figure 1c [32,36]. Our dynamic light scattering (DLS) studies showed that the average particle size of DNPs was around 222.43 ± 1.150 nm as shown in Table 1 and Figure 2a. The zeta potential of the nanoformulation was found to be −30.63 ± 1.365 mV with a poly dispersity index of 0.133 ± 0.014 as shown in Figure 2b and Table 1, respectively. We believe that low poly dispersity and particle size of around 222 nm may be attributed to the ethanol as desolvating agent and pH 8 used during the synthesis of the nanoparticles [37,38]. Moreover, our synthesized nanoformulation shows similar DLS and particle size distribution characteristics as shown in previously reported cases of BSA NPs [32]. The particle size and spherical morphology of the synthesized DNPs were also confirmed by SEM and TEM as shown in Figure 2c,d. Further, the drug entrapment studies show that the DNPs has encapsulation efficiency of 48.22 ± 1.948% and drug-loading of 4.28 ± 1.94 [39]. To evaluate and confirm the probable drug–protein interaction mechanism we performed molecular simulations and fluorescent quenching studies.

### 3.2. BSA Luminespib Interaction Studies

**Figure 3:**
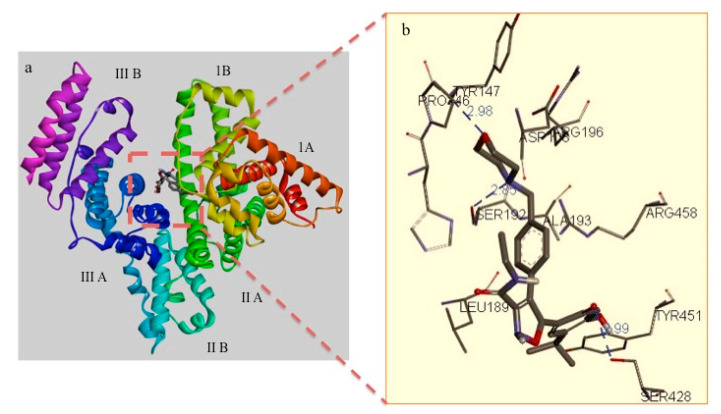
(**a**) Binding pocket for luminespib in BSA; (**b**) amino acid residues of BSA that interact with luminespib through hydrogen bond interactions. It also shows the interaction bonding distance for the hydrogen bonds when BSA protein interacts with luminespib.

Synthesis of BSA luminespib NPs depends on the interaction of luminespib with BSA at the structural level. To evaluate this as a first step, we performed luminespib docking studies to find the probable binding site. Our result indicates that Gibbs free energy of drug binding to BSA was −10.48 kcal/mol with frequency of 50% and predicted inhibition (*K_i_*) as 20.64 nM as shown in Figure 3a. The model shows that luminespib may form relatively more stable drug-BSA complex compared to previously reported cases of drug-BSA interaction due to relatively low binding free energy found in the range of −4.35 to −5.45 kcal/mol [32,40]. We also observed that the drug interacts with nearly fourteen interaction residues with mainly three important types of interactions such as H-bonds, polar and hydrophobic interactions. Our model indicates that the probable binding pocket of luminespib shows three hydrogen bond interactions, five polar and six hydrophobic interactions with BSA residues (as shown in Table 2). From the docking simulation, we believe that the probable binding site for the luminespib is hydrophilic domain I of BSA, Figure 3a. The stable hydrogen bond interaction between amino acid residues such as Tyr 147, Ser 428 and Ser 192 and luminespib is shown in Figure 3b. This may be due to the anionic nature of luminespib that helps in forming hydrogen bond interactions. In addition, it also has amine-containing heterocyclic functional group to stabilize the protein-ligand complex via. hydrogen bond interactions as seen with other pharmacophores [41,42].

**Figure 4:**
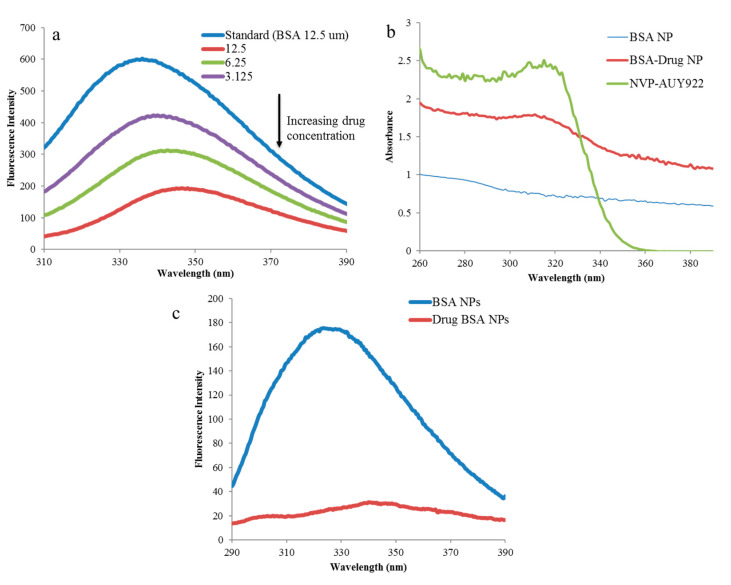
(**a**) Fluorescence quenching when BSA interacts with luminespib while formation of drug BSA nanoconjugates; (**b**) DNPs when subjected to UV absorptions studies, it shows presence of a peak around 310 nm, the same as that of the native drug and blank BSA NPs as controls; (**c**) DNP nanodispersion in PBS 7.4 pH at 1 mg/mL concentration was studied with fluorescence spectroscopy where native BSA NPs were taken as control.

In order to confirm that the drug interacts with the BSA, we performed fluorescence quenching experiment. Here, we kept the concentration of BSA as a constant (12.5 µM) and stepwise increased the concentration of luminespib solution (3.125 µM to 12.5 µM). We observed a great drop or quenching in the fluorescent intensity with the increase in the drug concentration as shown in Figure 4a. This indicates that the drug interacts with BSA, and quenching is attributed to the changes in the BSA microenvironment due to the drug interaction [43]. We also recorded the fluorescence spectra for native BSA NPs and compared it to that of DNPs at a concentration of 1 mg/mL. It was observed a nearly 8-fold difference as shown in Figure 4c. The decrease in the relative fluorescence intensity for DNPs can be attributed to the formation of drug-BSA complex [32]. The presence of the drug-BSA complex was also confirmed from the UV absorption peaks as shown in Figure 4b. The drug signal at around 310 nm in DNPs that coincides with pure drug signal as positive control was observed. The signal was absent in native BSA NPs. Overall, UV and fluorescence spectroscopy confirms the interaction between ligand and protein. Luminespib probably interacts with BSA through various non-covalent forms of interactions (indicated in Table 2) for the formation of self-assembled DNPs.

### 3.3. X-ray Photo Electron Spectroscopy, TLC and Stability Studies

**Figure 5:**
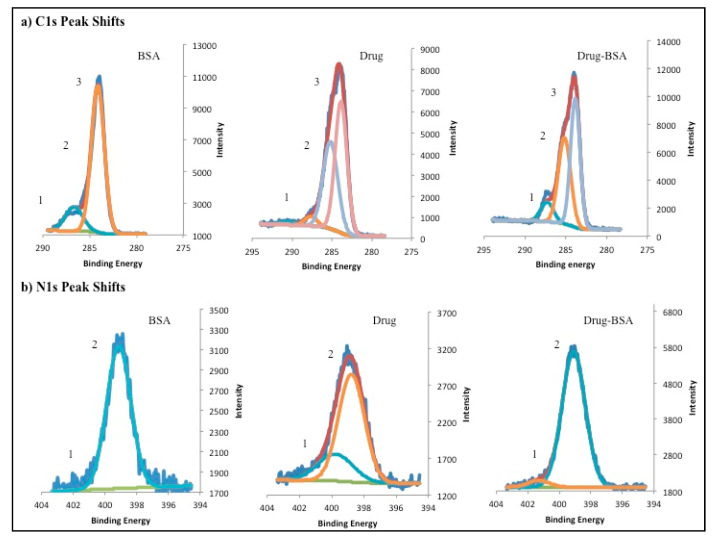
XPS spectra for carbon 1s (C 1s) and nitrogen 1s (N 1s) alterations in binding energy peaks for DNPs in comparison to native BSA NP and luminespib as control. (**a**) C 1s shifts also mark the presence of peak at second position for drug and DNPs whereas its absence in native BSA NPs; (**b**) N 1s peak shifts and the presence of peak at position one for drug and DNPs whereas its absence in native BSA NPs. Peak fitting function by multipack software, helps in identification of multiple peaks in the raw data. The sharp solid lines in above spectra represents peak fits for identification of single or multiple peaks in the wavy lines of raw data.

Further, to confirm the presence of the drug interaction with BSA we performed XPS analysis. It can be seen from Figure 1a that the drug consists of nitrogen- and oxygen- containing heterocyclic ring structure. Hence, the presence of these ring systems in DNP conjugates may provide relative shifts or changes in the atomic signals for carbon and nitrogen due to the electron-withdrawing and donating microenvironment of DNPs in comparison to native BSA NPs. Figure 5a shows the comparison of carbon signatures from DNPs, native BSA NPs and drug. It was observed that –N–C=O bond peak was observed at 286.45 for BSA, which was shifted to 287 (shifted by 1.05 eV) for DNPs. This positive shift may be attributed to the presence of electron withdrawing aromatic nucleus of the drug present in DNPs. Further, we also observed three-peak signatures for DNPs and drug sample that was absent in native BSA NPs as shown in Figure 5a. In this three-peak signature, the peak at 285 eV (Figure 5a) is correspondent to –C=N bond, which corresponds to the –C=N– of iso-oxazole moiety of the drug molecule (Figure 1a) [44].

Moreover, to understand the nature of changes in carbon signals, we analyzed the relative changes in the nitrogen signatures. It was observed that XPS peak for =N–O– was absent in native BSA, but it was present in drug and DNP spectra as shown in Figure 5b. The =N–O– peak was found to be slightly shifted to 401.3 for DNPs than native drug where it was found to be at 399.75 eV [45]. These results also suggest the drug-protein interactions, which is consistent with the fluorescence and UV.

**Figure 6:**
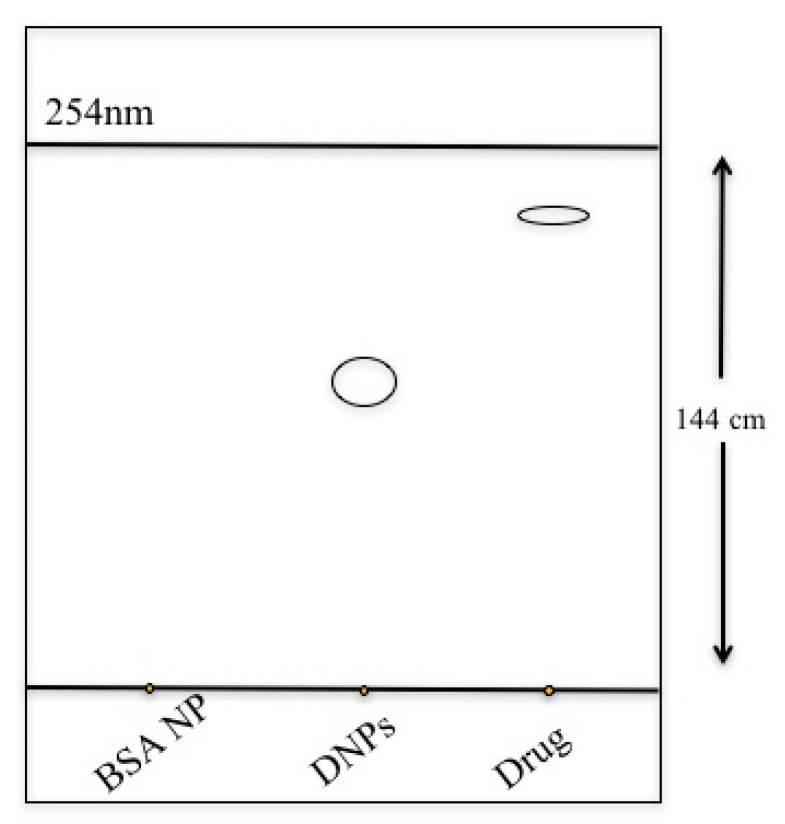
Thin-layer chromatography (TLC) image with the 10 µL spot of DNPs in comparison to native BSA NPs and luminespib on TLC plate as controls. The solvent front was run for 144 cm to calculate *R*_f_ values for respective mobility of spots for BSA NPs, DNPs and native drug. Our results show a real time shifts in the spot position for luminespib from DNPs in comparison to native drug. The calculated *R*_f_ values were performed n = 3 times and average were taken to determine the shift in the position of spots.

Furthermore, to confirm the presence of drug in DNP nanoconjugates, we performed TLC studies in comparison to native drug and BSA NPs as shown in Figure 6. It was observed that 10 µL spot of DNPs at 1 mg/mL concentration in deionized water provided an average *R*_f_ value of 0.64 ± 0.03. However, ethanol solution of native drug gave *R*_f_ value of 0.85 ± 0.005 (as shown in Figure 6). These observations clearly show the presence of drug in DNPs in comparison to positive (native drug) and negative (BSA NPs) control [31]. The difference in the *R*_f_ values for the native drug and DNPs is due to the interaction of drug with BSA in DNP formulation. This result also supported our previous experimental results provided by fluorescence and UV spectroscopy.

**Figure 7:**
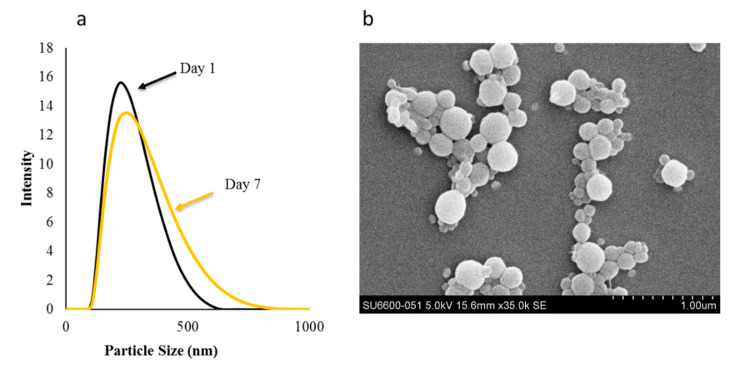
(**a**) Particle size distribution and (**b**) SEM image of the nanoparticles stored at 25 °C for 7 days.

It was also important to check the stability of the formulation to understand the storage condition of the formulation. Most nanoparticles preparations can be stored under room temperature [46,47]. To assess this, we performed stability evaluation for our preparation. As shown in Figure 7a,b. There was insignificant increase in the particle size from 220 nm (Day 0) to 255 nm (Day 7) and polydispersity index from 0.133 (Day 0) to 0.176 (Day 7). Further, we also observed that there was relative increase in number of particles in the size range of around 500 to 700 nm. This could be due to the polymer swelling over time or due to particle aggregations. To check the visual observation, SEM was performed. Overall, data from particle size distribution and SEM suggests that the nanoparticles are stable at room temperature for nearly 7 days, but may aggregated over time at 25 °C. Hence, dispersions require necessary sonication before use.

### 3.4. In Vitro Drug Release Studies

**Figure 8:**
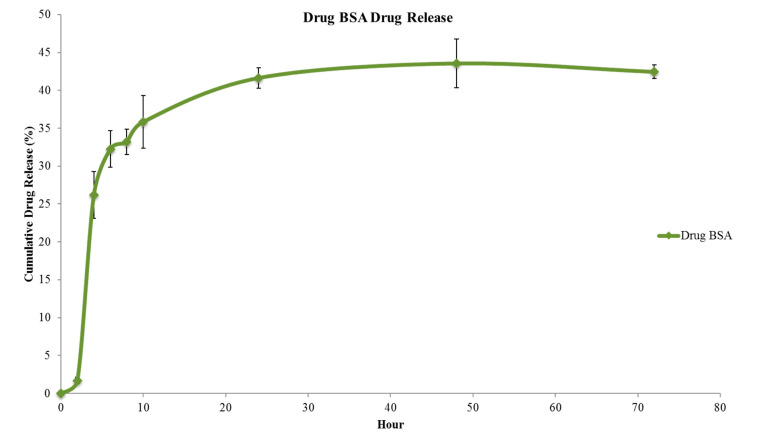
Time-dependent sustained release of drug from DNPs over a period of 72 h.

The drug release studies of DNP formulation were performed under pH 7.4 for 72 h. It was observed that the first burst release of the drug occurred in the first 8 h of incubation period. It can be seen that there was a time-dependent exponential increase in the drug release for 24 h. This was followed by sustained release of drug from DNP as shown in Figure 8. This study clearly shows that we may expect to see the desired pharmacological activity within 24 to 48 h.

### 3.5. In Vitro Cytotoxicity Studies

**Figure 9:**
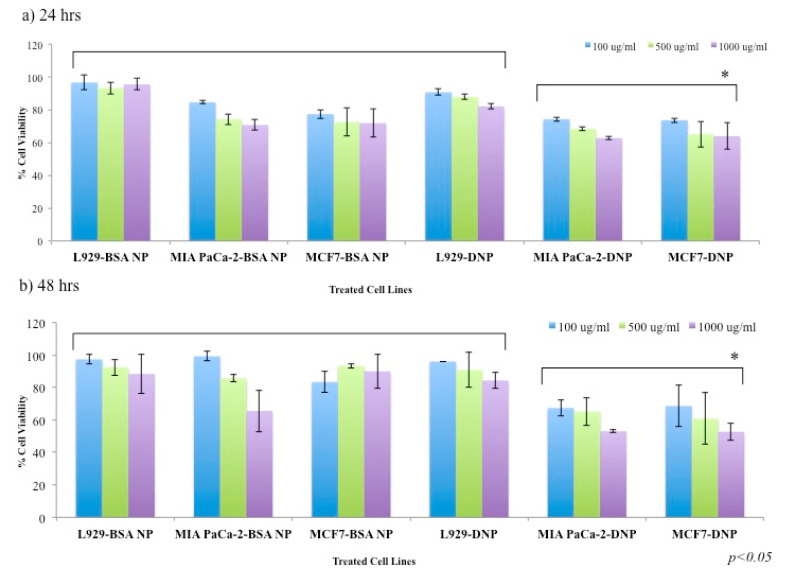
(**a**,**b**) Percent cell viability of MIA PaCa-2 and MCF-7 (cancer) cell lines treated with DNPs in comparison to treatment of L929 as control. Figure shows comparative account for cell lines treated with blank BSA NPs and DNPs. It also shows the cytotoxic and biocompatible effect exhibited by DNPs and native BSA NPs, respectively, in both concentration and a time-dependent manner. We observed that blank BSA NPs and DNPs are biocompatible towards L929 in a concentration- dependent manner compared to that of MIA PaCa-2 and MCF-7 with * *p* < 0.05. The comparison was drawn between groups (between test and control groups as well as between two time points) for calculation of statistical significance.

**Figure 10:**
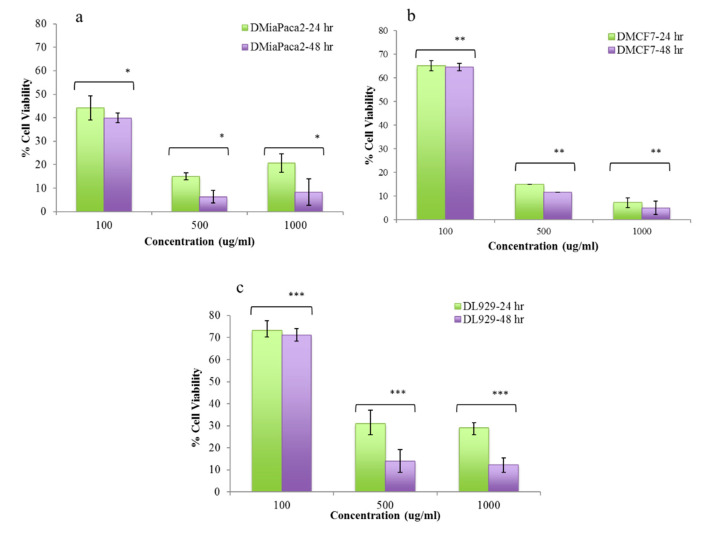
(**a**–**c**) Percent relative cytotoxicity of native luminespib in concentration and time-dependent manner towards MiaPaCa-2, MCF-7 and L929 cell lines. The study shows statistical significance of *, **, *** *p* < 0.05 within various groups marked in figure. The student *t* test carried out by comparing the lower concentration (100 µg/mL) treated group with high concentration (500 and 1000 µg/mL) treated group for each cell line.

To evaluate the anticancer therapeutic efficacy of synthesized DNPs, cytotoxicity studies were performed on cancer cell lines such as MIA PaCa-2 and MCF-7. We also checked the effect of DNPs on normal mice fibroblast (L929) cell line. Both, BSA NP treatment for MCF-7, MIA PaCA-2, L929 and DNP against L929 were taken as *−ve* controls for the analysis. In addition, native drug treated MCF-7, MIA PaCa-2 and L929 were taken as *+ve* control for the cytotoxicity assay (Figure 10). The cytotoxicity for all the test and *−ve* control group was recorded at 24 and 48 h intervals as shown in Figure 9.

We were able to see a concentration dependent cytotoxicity for DNPs towards MIA PaCa-2 and MCF-7 cell line in comparison to L929 as shown in Figure 9a,b (with *p* < 0.05). Moreover, a similarity in the therapeutic efficacy of DNPs formulation towards MIA PaCa-2 and MCF-7 cell lines for all the concentrations (0.1 to 1 mg/mL) was also observed, as shown in Figure 9a,b. At, 24 and 48 h of incubation for all the three cell lines, DNPs exhibited relatively high cytotoxicity towards cancer cell lines (MIA PaCa-2 and MCF-7) compared to normal L929 cell line.

Moreover, it was also observed that for MIA PaCa-2 and MCF-7 cell lines, DNPs shows good cell growth inhibition at 1 mg/mL concentration in 24 h and 48 h intervals (*p < 0.05*) as shown in Figure 9. After 24 h of incubation we observed that DNPs at 1 mg/mL concentration, the percentage of cell viability for MIA PaCa-2 and MCF-7 cells were found to be around 62% and 63%, respectively. After 48 h, the viability further dropped to around 53% and 52% for MIA PaCa-2 and MCF-7, respectively. A similar variation in percentage of cell viability of MIA PaCa-2 and MCF-7 was seen for other concentrations of DNPs (with *p* < 0.05) as shown in Figure 9. Our results clearly shows selective cancer killing by DNPs due to the presence of Hsp90 inhibitor (luminespib) in them [22]. In comparison to standard free drug (luminespib), DNPs formulations were shown to have controlled and sustained cytotoxic (over 24 and 48 h) effect in comparison to free drug (Figure 10) for all the three concentrations against all the three cell lines.

Our results about the biocompatibility of BSA encapsulated drugs formulation and bare BSA NPs against MIA PaCa-2, MCF-7 and L929 align with previously reported studies about the BSA conjugated nanoformulations [25,32,48] as shown in Figure 1b and Figure 9. Hence, aqueous dispensable luminespib-loaded BSA NPs may prove to be an important step towards the use of this formulation for its anticancer effect due to Hsp90 inhibition against breast cancer and pancreatic cancer. The representative Figure 1c and cytotoxicity assay in Figure 9a,b clearly show anticancer behavior of our DNP formulation against cancer cell lines, which may be attributed to drug release over a period of time (shown in Figure 8). These results clearly show that our aqueous dispensable DNP formulation may prove to be a viable option for breast cancer and pancreatic cancer therapy.

## 4. Conclusions

We have developed luminespib-loaded BSA NPs (DNPs) for breast and pancreatic cancer therapy that can be dispensed in an aqueous vehicle. Our fluorescence quenching experiments show that luminespib interacts with BSA and forms nanoparticles by classical desolvation method. Our docking study predicted that the probable binding site of the luminespib in BSA during formation of DNPs with lowest Gibbs free energy of binding of drug-BSA complex is around hydrophilic domain I of BSA. Our cellular biocompatibility studies also demonstrate the safety profile in a concentration-dependent manner for DNPs and native BSA NPs in normal L929 cell line in comparison to cancer cell lines such as MIA PaCa-2 and MCF-7. We also observed significant (*p* < 0.05) anticancer effect for our DNPs in comparison to native BSA NPs. We believe that this difference is due to non-covalent conjugation of drug in DNPs in comparison to native BSA NPs. We found that the anticancer efficacy of our DNP formulation was similar for MIA PaCa-2 and MCF-7 cell lines. We believe that this work can be the first step in the direction of improving the formulation Hsp90 inhibitors by using natural biomaterials like BSA.

## Figures and Tables

**Table 1:** Physical-chemical characterization of drug-loaded BSA NPs (DNPs).

Parameters	Values
Z-average particle size (nm)	222.433 ± 1.150
Zeta potential average (mV)	−30.63 ± 1.365
Poly dispersity index (PDI)	0.133 ± 0.014
Encapsulation efficiency (%)	48.22 ± 1.948
Drug loading	4.28 ± 1.94

**Table 2:** The amino acid residues of BSA (PDI: 3V03) that interacts with luminespib to form a stable protein-ligand complex.

Hydrogen Bond	Polar	Cation-pi	Hydrophobic	Other
TYR147 (−0.6974)	ARG458 (−1.526)	TYR451 (−0.6994)	ALA193 (−0.5145)	LEU189 (−1.6106)
SER428 (−0.04736)	HIS145 (−1.1971)		PRO146 (−0.4804)	THR190 (−0.6233)
SER192 (−0.4233)	ARG196 (−1.0906)		ILE455 (−0.4311)	
	GLU424 (−0.5991)			
	ASP108 (−0.479)			
